# A Multicenter Experience of the Treatment of Type Di Tympanojugular Paragangliomas: Total vs. Partial Resection and Residue Management

**DOI:** 10.3390/jcm14186579

**Published:** 2025-09-18

**Authors:** Daniele Marchioni, Nicola Bisi, Mohamed Badr-El-Dine, George Wanna, Zachary G. Schwam, Mohamed Fawzy Fathalla, Alessia Rubini

**Affiliations:** 1 Department of Otolaryngology-Head and Neck Surgery, University Hospital of Modena, 41125 Modena, Italy; 2 Department of Otolaryngology, Faculty of Medicine, Alexandria University, Alexandria 21526, Egypt; mbeldine@yahoo.com; 3 Department of Otolaryngology-Head and Neck Surgery, Icahn School of Medicine at Mount Sinai, New York, NY 10029, USA; 4 Department of Otorhinolaryngology-Head & Neck Surgery, Faculty of Medicine, Suez University, Suez 41522, Egypt

**Keywords:** type Di tympanojugular paragangliomas, infratemporal fossa type A approach, paraganglioma

## Abstract

**Introduction**: Tympanojugular paragangliomas (TJ-PGs) showing intradural growth into the cerebellopontine angle (Fisch classification Di) represent a surgical challenge, with their proper surgical management still under debate. **Methods**: This is an international multicenter retrospective review of patients with Di TJ-PGs who underwent surgery in three high-volume skull base surgery centers. We aimed to establish practice patterns for treating Di TJ-PGs, namely the surgical approach, total versus partial resection, and whether a staged procedure was needed. We also examined the status of the facial and lower cranial nerves (LCNs), postoperative complications, and residue management after partial resection. **Results**: Thirty-two patients were included in this study with an average follow-up of 66 months. Preoperative angiography with selective embolization was performed in all patients, and a type A infratemporal fossa approach was the most common surgical technique. Total resection was achieved in 16 cases. A single-stage procedure was performed in 26 patients and a staged procedure in 6. CSF leakage in the neck was the main reported complication. Most patients had an HB I-II-grade facial nerve at the last follow-up, and three patients experienced worsened lower cranial neuropathies. In 16 patients residual disease was present after surgery and was managed with either radiotherapy or observation. **Conclusions**: Di TJ-PGs pose a complex treatment challenge for which clear-cut management recommendations have not been established. Surgical resection, when indicated, may be total, the preferred option in young healthy candidates, or partial, mainly employed in elderly or high-risk patients, always considering the tumor’s relationship to critical structures. When residual tumor is present, both radiological surveillance and adjuvant radiotherapy can be effective strategies.

## 1. Introduction

Tympanojugular paragangliomas (TJ-PGs) are typically benign, hypervascular, non-secreting, indolent tumors that originate from the paraganglion cells of the jugular bulb, arise within the jugular foramen, and grow into the temporal bone, neck, and endocranium.

Although benign, TJ-PGs may manifest late, with considerable morbidity. Their progression throughout the skull base can penetrate pneumatized air cell tracts, vascular channels, and foramina, and often causes IX, X, and XI cranial nerve dysfunction [1].

**Figure 1:**
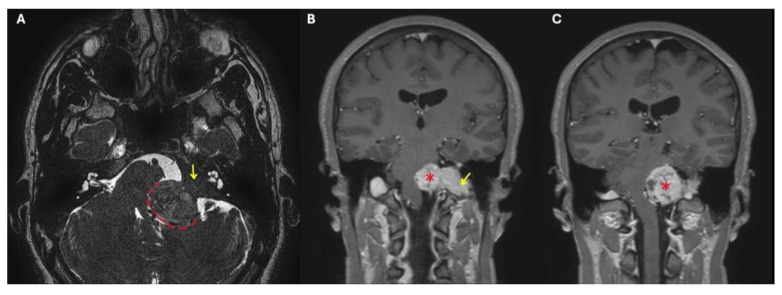
Brain and cerebellopontine-angle MRI of a TJ-PG Di2 class. (**A**): T2-weighted image; (**B**) and (**C**): T1-weighted images with contrast enhancement. *: intradural portion of the TJ-PG Di2; dashed line: brainstem compression by the TJ-PG Di2 in the cisterna magna; yellow arrow: extradural portion of the TJ-PG Di2.

Fisch classified TJ-PGs as types A-D in order of increasing disease extent. This classification considers that up to 20% of these tumors may develop intracranial extension and that their progression can be extradural or intradural. In the latter case, tumors with ≤2 cm of intradural growth are defined as Di1 lesions, while those with a >2 cm extension are called Di2 (Figure 1) [2].

Only in very rare cases of massive intradural growth do TJ-PGs present with symptomatic hydrocephalus or brainstem compression [3].

Managing Di TJ-PGs is rather challenging and controversial at times, and requires a multidisciplinary approach. Microsurgical resection is one of the mainstays of treatment; resection can be partial or total, and may occur in one or two stages (often resecting the extracranial component first)

Stereotactic radiosurgery (SRS) also has a relevant role, alone or in combination with microsurgical resection. Primary SRS may achieve local tumor control in up to 96% of type C and D TJ-PGs, but since it often leads to temporary tumoral edema, it should be avoided in patients with Di lesions and brainstem compression. It is also important to note that surgery following primary SRS is more challenging and more often leads to morbidity [4].

Careful observation through clinical examination and serial imaging represents an option in elderly patients with stable radiographic disease, minimal symptoms, and no signs of brainstem compression. Thanks to advancements in both imaging and surgical techniques, even very large intradural TJ-PGs, previously considered inoperable, can now be aggressively managed surgically with an acceptable morbidity profile [5].

**Figure 2:**
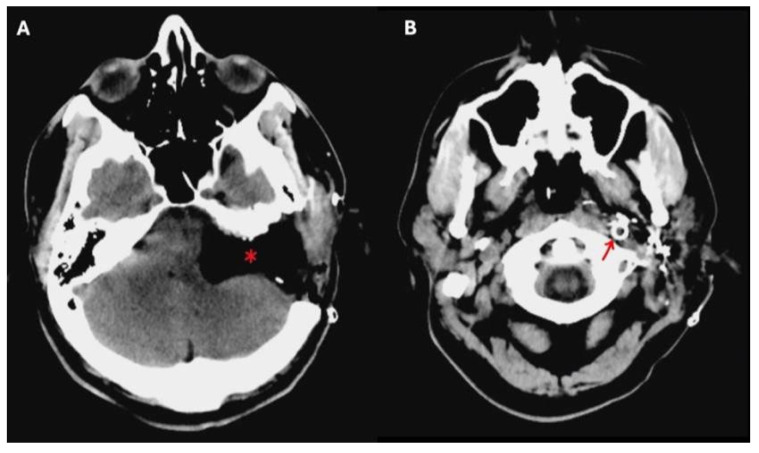
A postoperative brain CT scan after total removal of a TJ-PG Di2 in a single-stage procedure. In (**A**), * indicates the surgical cavity after the total removal of the TJ-PG Di2. In (**B**), the red arrow indicates the endovascular stent placed preoperatively in the internal carotid artery.

Preoperative angiography has played a vital role in allowing these kinds of large surgical resections, since it gives information concerning the vascularity of the tumor, and the involvement of the internal carotid artery (ICA) and the tumor-feeding vessels, also allowing for their selective embolization. Balloon occlusion testing can inform the risk of stroke should the ICA be sacrificed during the procedure, and an endovascular stent in a tumor-involved vertical petrous ICA can be placed for safer tumor dissection from the vessel walls (Figure 2) [6].

Surgical resection of Di TJ-PGs is extremely demanding for the surgical team, and the shared expertise of both the neurotologist and the neurosurgeon is paramount for proper resection with preservation of vital structures such as the ICA, the facial nerve (FN), and the functioning LCNs.

Lower cranial neuropathies often signal the presence of intradural disease, as one of the most common paths of early intradural extension is via the medial wall of the jugular bulb [7].

The current paper stems from the lack of guidelines and consensus for management of intradural TJ-PGs. There is no consensus in the surgical community as to whether a partial or total resection is to be carried out or what to do in the management of residual disease. Other discussion points concern whether staging is preferred and the preoperative handling of the ICA.

The authors deemed it necessary to share their expertise in this multicenter work with the aim of presenting one of the largest case series on the matter and attempting to propose some recommendations from their experience.

## 2. Materials and Methods

A retrospective multicenter analysis was conducted to evaluate the surgical outcomes of TJ-PG patients with intradural extension (Fisch Classification Class Di), treated between January 2014 and December 2024. The participating centers included the University Hospital of Modena (Italy), the University Hospital of Alexandria (Egypt), and the Mount Sinai Health System (USA).

Only patients diagnosed with Di-class TJ-PG who underwent surgical resection were included in our study, while those who were treated with non-surgical approaches were excluded. Patients with multifocal disease were excluded.

All included tumors were classified according to the Fisch tumor classification, initially based on preoperative imaging, and subsequently confirmed by intraoperative findings [8].

Both the preoperative management of the internal ICA and the surgical approach adopted by each center were recorded. The former was described including either endovascular stenting or therapeutic vessel occlusion, depending on the individual case and the vascular involvement.

Particular attention was also paid to the dissection of the tumor from the intrapetrous ICA, brainstem, cavernous sinus, and cerebellopontine-angle structures.

The surgical technique was further subdivided based on whether the intradural and extradural components were excised concomitantly or in a staged approach.

Tumor resections were categorized into two groups according to the intraoperative records:

- Total resection: This is defined as the complete macroscopic removal of the tumor with no visible residual in the surgical field.
- Partial resection: This is defined as intentionally leaving tumor remnants in critical areas such as around the intrapetrous ICA, cavernous sinus, brainstem, and LCNs. Preoperative and postoperative evaluations included the hearing and FN function, as well as the status of IX-XII.

Postoperative cranial nerve function was evaluated at 6-month follow up.

Postoperative FN function in patients who presented with normal preoperative FN function was categorized according to the House–Brackmann scale into the following groups: Grade I–II, Grade III– IV, and Grade V–VI.

Data on the follow-up, postoperative complications, and any radiological evidence of residual tumor were also collected. In cases where follow-up MRI revealed residual disease, the subsequent chosen management strategy (e.g., observation, radiotherapy) was documented accordingly.

## 3. Results

A total of 32 patients with Di-class TJ-PGs were included in the study (19 female and 13 male subjects). The mean age of the patients at the time of surgery was 46.6 years (range: 23–65), while the mean radiologic follow-up time was 66.3 months (range: 6–240 months) (Table 1).

### 3.1. Classification

**Figure 3:**
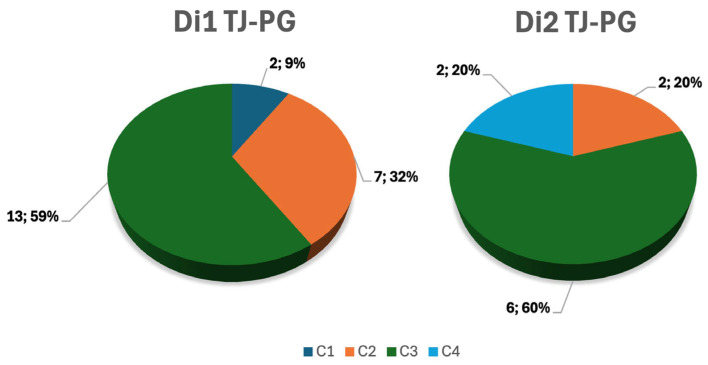
Pie charts showing the percentage of each Fisch C-category classification in two groups of patients identified according to the grade of intradural extension of the tympanojugular paraganglioma (Di1 and Di2).

Depending on the preoperative imaging (temporal bone CT scan, MRI, or angiography), the tumors were classified according to Fisch Classification [2] as follows:

2/32 C1 class; 9/32 C2; 19/32 C3; 2/32 C4. For what concerns the D Class, 22/32 belonged to the Di1 class and 10/23 to Di2. Further subdividing these last two groups, of the Di1 group, 2/22 were C1, 7/22 were C2, and 13/22 were C3. On the other hand, for the Di2 class, 2/10 were C2; 6/10 were C3; and 2/10 were C4 (Figure 3).

### 3.2. Preoperative Management of the ICA

Angiography with balloon test occlusion of the ICA was performed in all patients.

In 8/32 patients pre-surgical stenting of the ICA was performed given the involvement of the wall of the vertical intrapetrous portion. After stenting, the patients required dual antiplatelet therapy for two months before the planned surgery.

In 2/32 patients with class C4-Di2 and C3-Di2 TJ-PGs tumors, permanent carotid occlusion was performed; adequate collaterals were confirmed before doing so.

In 22/32 cases, no ICA treatment was performed before surgery.

In all cases (32/32 patients), angiography with embolization of the tumor’s blood supply was carried out 24–48 h before surgery.

### 3.3. Surgical Approaches

Staged surgery was performed in 6/32 patients, while a single-stage procedure was adopted in 26/32 patients. A Type A infratemporal fossa approach was the primary choice (15/32), while an adjunctive translabyrinthine approach was required in 13/32, an adjunctive transcochlear approach in 3/32, and an adjunctive far lateral approach in 1/32.

A second-stage procedure was necessary in 6/32 to remove the intradural portion of the tumor. A retrosigmoid approach was performed in 3/32, and a trans-dural trans-sigmoid trans-condylar approach in the other 3.

### 3.4. Dural Repair in the Single-Stage Procedure

In 26/32 patients a single-stage surgical procedure was adopted in order to remove the tumor. In 25/26 a fat graft was used to fill the dural defect, and temporalis/sternocleidomastoid muscle rotational flaps were used to separate the temporal cavity from the neck.

A free flap was necessary in 1/26 to cover a very large defect.

### 3.5. Total Versus Partial Resection

**Figure 4:**
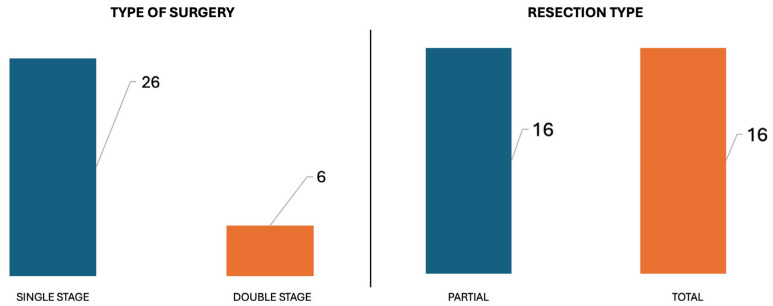
Histograms displaying, at a glance, the proportion of patients according to the type of surgery (single vs. double stage) and the degree of resection (partial vs. total).

In 16/32 patients it was possible to achieve total tumor removal, while in the other 16 a partial resection was performed (Figure 4).

In the total resection group, the majority (14/16) were class Di1 tumors (two C1Di1; five C2Di1; seven C3Di1), and two were C3Di2 class tumors. Preoperative carotid stenting was performed in 6/16 cases.

In those who underwent a partial tumor removal (16/32), 12/16 required no treatment of the carotid artery, while 2/16 required a stent and 2/16 required endovascular carotid sacrifice. Residual tumor was left on critical structures including the cavernous sinus (5/16), vertical petrous carotid (6/16), horizontal petrous carotid/petrous carotid (2/16), and lower cranial nerves (1/16).

### 3.6. Hearing Function

Profound sensorineural hearing loss ipsilateral to the tumor was found before surgery in 4/32 patients due to inner ear tumoral involvement.

Conductive or mixed hearing loss was observed preoperatively in 28/32 cases; 13 of these subsequently had profound hearing loss postoperatively.

### 3.7. Facial Nerve Outcome

Facial nerve function was normal preop (HB 1) in 18/32 cases. Of these, postoperatively, 11/18 had HB I-II, 3/18 had III-IV, and 4/18 had HB V-VI. Preoperatively, six patients had HB V-VI facial palsy. In these cases, the facial nerve was resected; a concomitant cable graft was placed in three patients, and later facial reanimation performed in the remainder.

### 3.8. Lower Cranial Nerves and Hypoglossal Nerve

With respect to lower cranial nerve function, 22/32 patients had preop dysfunction, while 10 were neurologically intact. In patients with normal nerve function, a small residual tumor was left on the nerves. In 7/10 the nerve function was preserved. Hypoglossal nerve function was normal in 27/32 patients preop and preserved in 26/27.

### 3.9. Residual Disease Management

In 16/32 patients residual disease was present; 9/16 underwent subsequent adjuvant SRS, and 7/16 were managed expectantly with observation and serial MRIs. Of these 7, 3 demonstrated progressive tumor growth. These were all radiated with SRS. Salvage surgery was not required in any patient.

### 3.10. Complications

No intraoperative complications, and, in particular, no ICA ruptures, occurred in our series. A postoperative CSF leak was found in 4/32 patients; 3 of these patients underwent single-stage surgery. Only 1/4 required revision surgery. There were two deaths; one from meningitis and the other from aspiration pneumonia after lower nerve palsy.

## 4. Discussion

TJ-PGs originate from the paraganglia associated with the adventitia of the jugular bulb. Their staging and classification depend on temporal bone infiltration, carotid canal involvement, intracranial and intradural extension, and vertebral artery infiltration. The 1988 Fisch classification, which categorizes tumors based on their extension along the intrapetrous internal carotid artery, has long been widely accepted. A thorough understanding of the pathological and anatomical characteristics of these tumors, especially the Di class with intradural extension, is essential for clinical decision-making and particularly relevant to the objectives of our study, given the distinct challenges this subgroup presents. Di TJ-PGs are insidiously growing tumors showing aggressive behavior; they often demonstrate extensive erosion of the temporal bone and lateral skull base, infiltration of the LCNs, encasement of the ICA, and extension into the CPA. Intradural invasion of the CPA may occur through penetration of the posterior fossa dura along the LCNs or via the internal auditory canal and the dura of the middle cranial fossa [7,9].

Some studies show that the higher the C class of TJ-PGs is, the greater the likelihood of intradural invasion, since involvement of the ICA seems to directly correlate with the degree of intradural extension [3,7].

Our results confirm the correlation between the intrapetrous carotid artery encasement and the degree of CPA extension of the tumor. In only 2/32 cases we observed partial encasement of the intrapetrous vertical portion of the internal carotid artery (Class C1-Di) in association with intradural extension of the tumor. This could be explained by the fact that in these cases, the tumor grew along the pars nervosa portion of the jugular foramen following the LCNs, infiltrating the medial wall of the jugular bulb and reaching the cerebellopontine angle.

This tumor behavior may help explain why patients with Di tumors often present with LCN involvement.

In our series, the most common presenting symptoms were pulsatile tinnitus, conductive hearing loss, otalgia, and aural fullness, clinical features that are typical across all types of TJ-PGs. Additional symptoms often depend on tumor size and the extent of extension. Tumors with intradural extension frequently involve LCN deficits, manifesting as dysphagia, hoarseness, aspiration, shoulder drop, and tongue deviation. In cases of significant intracranial progression, compression of the brainstem and cerebellum may occur, leading to cerebellar signs, hydrocephalus, increased intracranial pressure, gait instability, and, in severe cases, coma.

The treatment of TJ-PGs generally comprises three options: the wait-and-scan policy, surgery, and radiotherapy. Treatment is highly individualized depending on tumor class, location, patient comorbidities, and preference.

As far as radiotherapy for Class Di tumors is concerned, some considerations should be made. In Di2 tumors with considerable brainstem compression, significant edema may result in worsening compression. Additionally, surgery after radiotherapy could be more difficult, and intraoperative complications are to be expected. For this reason, in Di TJ-PG radiotherapy (large-volume or stereotactic radiation delivery) should be limited to postoperative management in cases of residual progressive disease. As a first choice of treatment, stereotactic radiosurgery may be used in slow-growing tumors in the elderly and in patients with considerable comorbidities in order to achieve a high local tumor control with limited morbidity.

“Watchful waiting” remains a viable option in elderly patients with stable tumors during radiological follow-up.

Regarding the surgical management of intradural TJ-PG, this must be meticulously planned, taking into account several critical factors: the patient’s age and comorbidities, the functional status of the ipsilateral and contralateral LCNs, hypoglossal nerve function, the extent of ICA encasement, potential brainstem invasion, and the tumor’s vascular supply, particularly from the brainstem and the posterior inferior cerebellar artery (PICA).

Even though there are few studies in the literature addressing the surgical management of these lesions, our multicenter study alone, supported by a thorough analysis of the existing works, allows for the proposal of several clinical recommendations. In younger and healthy patients, total resection should be the primary surgical objective whenever feasible, even though total resection at the expense of functioning LCNs or FN is still greatly debated in the literature. In contrast, in elderly patients or patients with a perioperative condition, and particularly in those presenting with encasement of critical neurovascular structures, cavernous sinus involvement, or intraoperative brainstem adhesion, a more conservative strategy like a partial resection followed by SRS is recommended, due to their significantly increased risk of surgical morbidity and mortality.

### 4.1. Preoperative Evaluations

For a proper preoperative assessment of Di tumors, an MRI evaluation of the intradural involvement of the CPA, the internal auditory canal, the LCNs, the clivus, the cavernous sinus, Meckel’s cave, the prepontine cistern, and the hypoglossal canal is of paramount importance. Accurate radiological staging is critical for planning the surgical approach and estimating any potential surgical morbidity.

In addition to MRI, the preoperative assessment of Di-type TJ-PG must include digital subtraction angiography to evaluate the tumor’s vascular supply. During the same procedure, balloon test occlusion (BTO) of the ICA is recommended to assess the adequacy of collateral cerebral circulation. This step is crucial in determining whether the ICA can be safely sacrificed intraoperatively in the event of complications such as arterial rupture or encasement needing vessel sacrifice.

When the preoperative MRI reveals more than 270° tumor encasement of the intrapetrous ICA or when angiography shows evidence of arterial stenosis or wall irregularities, interventional radiologic treatment is indicated. In particular, for tumors classified as Class C3 or C4, preoperative ICA stenting should be considered. This approach may reduce the risk of intraoperative arterial injury and allows for more aggressive dissection around the ICA to achieve tumor removal. Once a stent has been placed, a delay of 4 to 6 weeks before surgery is generally required to allow for the formation and stabilization of a neointimal lining on the luminal surface of the stent. However, in cases where unfavorable anatomical conditions, such as kinking or tortuosity of the cervical ICA, or total encasement with wall irregularities, preclude safe stent placement, and if good collateral circulation is confirmed via BTO, preoperative endovascular sacrifice of the ICA may be considered.

In our multicenter study, preoperative ICA stenting was performed in 8/32 patients, dependent on tumor classification. Additionally, permanent balloon occlusion of the intrapetrous ICA was carried out in two patients with Class C4-Di2 and Class C3-Di2 tumors, respectively. In the remaining 22 patients, no preoperative ICA intervention was performed. Notably, no intraoperative ICA wall injuries were observed in any of these patients, regardless of whether stenting or occlusion was performed. In particular, no statistically significant differences were observed between patients who underwent preoperative ICA stenting and those who underwent surgery without stenting, either in terms of intraoperative carotid artery rupture or the extent of tumor resection (total versus partial). Although several authors have recommended preoperative ICA stenting, there is currently no scientific evidence confirming the actual benefits of this procedure, especially when a partial resection is planned [10].

Finally, especially in Di TJ-PGs, a preoperative analysis of the relationship between the tumor and the brainstem is crucial in order to plan the surgical strategy. The larger the tumor and the closer the contact with the brainstem, the more important it is to study the possible intradural vascular contributions from PICA. In cases in which AICA/PICA/cavernous sinus encasement and brain invasion are expected, we advocate for partial resection.

### 4.2. Type of Surgery

**Figure 5:**
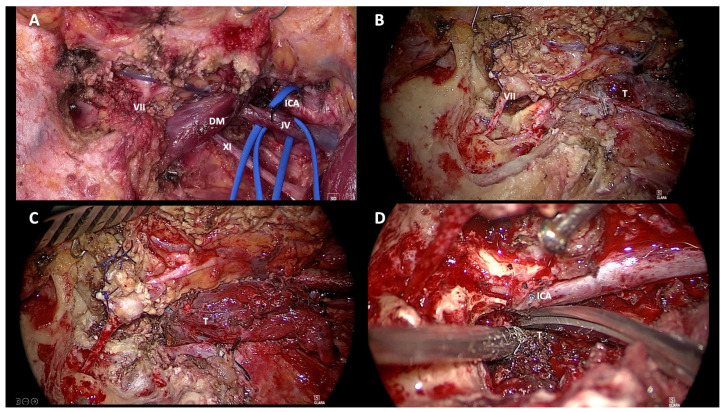
Surgical steps of the Fisch Type A Infratemporal Approach. (**A**): Isolation of the vessels in the neck (the internal carotid artery and the internal jugular vein). (**B**): Isolation of the intratemporal facial nerve and anterior rerouting. (**C**): Identification of the extracranial component of the TJ-TG. (**D**): Dissection of the tumor from the internal carotid artery in the neck and temporal bone. Endovascular stenting was performed before surgery. VII: VII cranial nerve; DM: posterior belly of digastric muscle; XI: XI cranial nerve; JV: jugular vein; ICA: internal carotid artery; T: tumor.

**Figure 6:**
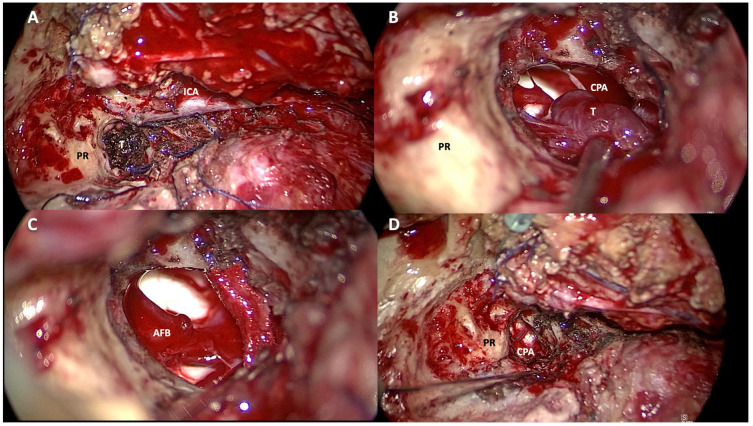
(**A**): Intradural portion of the tumor. (**B**): Removal of the component within the cerebellopontine angle. (**C**): View of the cerebellopontine angle with the acoustic-facial bundle after removal of the intradural tumor. (**D**): Final surgical cavity. PR: promontory; ICA: internal carotid artery; T: tumor; CPA: cerebellopontine angle; AFB: acoustic-facial bundle.

Choosing the most appropriate surgical approach in the management of patients with jugular foramen tumors is still a challenge. This becomes even more difficult when dealing with class Di TJ-PGs. In keeping with the Fisch classification, the surgical approach should be selected according to the tumor involvement along the carotid artery (Class C) and the extent of its intradural extension (Class Di). The surgical approaches adopted for the removal of Di TJ-PGs are variable in the literature and are often related to the experience of the surgeon. The infratemporal fossa approach type A (ITFA) described by Fisch in 1978 remains the most widely used approach for the removal of Di TJ-PGs (Figure 5 and Figure 6). [2]

In this technique the closure of the ear canal produces permanent complete conductive hearing loss, and the rerouting of the descending FN also causes permanent facial palsy (at least grade III according to the House–Brackmann scale), in patients without preoperative hearing loss or FN dysfunction.

In an effort to reduce surgical morbidity and maintain adequate exposure of the jugular foramen, some authors have also suggested the fallopian bridge technique for jugular foramen tumors with intracranial extension, a potentially less invasive option with better functional outcomes. In Oghalai and colleagues’ experience, a transjugular craniotomy with the fallopian bridge technique may also be adopted for class Di TJ-PGs, allowing for excellent exposure of both the intracranial and the extracranial components of the disease without FN mobilization, leading to an improved rate of FN preservation and avoiding closure of the external auditory canal [11].

When the extension of the tumor along the intrapetrous carotid artery is Class ≥ C2, in the authors’ experience, an ITFA should be adopted as a primary procedure, since anterior rerouting of the facial nerve is crucial to allow for infiltrated-bone removal around the jugular fossa and to remove the anterior extension of the tumor along the carotid canal, especially when total resection is attempted.

Given the possible morbidity of the ICA, LCNs, and FN caused by the total resection of TJ-PGs, in recent years, the skull base research community has strongly recommended partial tumor resection. This major paradigm shift emerged from the fact that TJ-PG is a slow-growing, benign tumor, and that surgery often causes several cranial nerve deficits with a large detriment to quality of life. We advocate for a careful assessment examining age, performance status, preoperative LCN status, and tumoral extent to determine whether a partial resection is most appropriate.

It is well known that class Di tumors in young patients show a particularly aggressive pattern, with a high recurrence rate during the follow-up time. Moreover, it is known that in young patients, even when iatrogenic FN and LCN deficits are expected, they are usually well tolerated and compensated for in the postoperative period [3,12].

For this reason, in cases of young patients with Di TJ-PGs, we advocate for total resection where possible, except in cases of unresectable disease, given these patients’ long life expectancy and the possibility of aggressive recurrences. In contrast, we advocate for partial resections in elderly patients with class Di TJ-PGs with serviceable LCNs given the devastating effects of iatrogenic neural deficits.

In our multicenter series, total tumor resection was achieved in 50% of cases, while in the remaining 50%, intentional residual tumor was left in anatomically and functionally critical areas to preserve neurological function.

In the presence of an intradural component, there are conflicting opinions in the literature on whether to perform a single-stage or two-stage procedure. This decision often depends on the extent of intradural involvement, the patient’s overall condition, and the surgeon’s experience. While a single-stage approach allows for complete tumor management in one session, a staged approach may reduce operative time, limit morbidity, and allow for neurological recovery between stages.

Some authors have suggested performing a single-stage resection of the paraganglioma regardless of the size of the intradural component of the tumor; however, this surgical solution requires careful reconstruction at the end of tumor excision [11,12,13,14].

At the end of single-stage surgery, a large cavity is created that directly connects the brainstem with the neck; sealing the lateral skull base and separating these two cavities is crucial.

Some authors claim that a staged procedure is preferable to a single operation to minimize the risk of postoperative CSF leak. Empirically, staged surgery tends to have lower risk to the LCNs. Accordingly, Jenkins and Fisch recommended a staged resection only for tumors with ≥2 cm intracranial extension [15], whereas Kinney recommended a two-stage resection with the intracranial component being removed first [16].

Sivalingam and colleagues recommended a staged approach with the intracranial component resected second; this was due to the devascularization of the intracranial component often found after the initial resection. They also recommended a second embolization before the staged component [17].

Several approaches are described in the literature concerning the second stage based on the experience of the operating center, on the hearing function of the patient, and on the location and size of the residual tumor. The modified transcochlear approach and the transdural–transsigmoid–transcondylar–transclival approach, as well as the retrosigmoid approach and the translabyrinthine and transotic techniques, are described in the literature and adopted in second-stage surgery.

Magliulo et al. employed the staged procedure to treat class Di TJ-PGs in particular using a first-stage type A ITFA with a second-stage retrosigmoid approach, with an 18% rate of CSF leak in the postoperative period [18].

Conversely, Patel et al. observed a 40% CSF leak in a cohort of patients treated with a staged procedure; in particular, they employed a first-stage transtemporal and preauricular subtemporal approach with a second-stage retrosigmoid/extreme lateral/transcondylar technique [3].

These relatively high rates of postoperative CSF leak underscore the importance of adequate dural closure and skull base reconstruction, particularly in complex multi-staged and multi-compartment procedures.

In the experience of Jackson, one-stage surgery is also preferable for jugular paragangliomas with intracranial extension since it offers the greatest likelihood of complete tumor removal and the best opportunity for CSF leak repair [9].

As far as the order of resection is concerned, we believe removing the extradural component first is advantageous to reduce the vascular supply. By devascularizing the tumor early, bleeding within the CPA can be minimized, thereby improving intraoperative visibility and reducing the risk of damage to neurovascular structures.

### 4.3. Skull Base Reconstruction

If resection is performed in a single stage, reconstruction of the dural defect should be tailored to the size and location of the defect. One must obliterate the dead space, often with abdominal fat and pedicled temporalis rotation flaps.

We often use temporalis and sternocleidomastoid muscle flaps, regardless of the size of the dural defect. Despite their limited arc of rotation, suturing of these muscles around the lateral skull base to separate the temporal bone cavity from the neck may facilitate skull base closure. On occasion, for very large tumors with correspondingly large skull base defects, a free flap may be needed to obliterate dead space and improve soft-tissue contouring.

In our study, there was no difference in the rates of CSF leak between staged and single-surgery cohorts. Of the twenty-six patients who underwent single-stage surgery, only four (15%) developed postoperative CSF collections in the neck; three cases were managed conservatively with compression dressings and diuretics, while one required revision surgery.

These preliminary findings suggest that single-stage surgery can achieve outcomes comparable to staged procedures, both in terms of postoperative CSF leak rate and tumor control. However, the success of this approach relies on meticulous lateral skull base reconstruction. Large cohort studies directly comparing CSF leakage rates between single-stage and two-stage procedures are essential to validate our assumptions.

### 4.4. Lower Cranial Nerves

As stated before, Di TJ-PGs may grow along the pars nervosa, infiltrating the medial wall of the jugular bulb before entering the CPA. If this happens, a LCN deficit is to be expected postoperatively given that dissection occurs medial to the jugular bulb [19].

When a patient has serviceable LCN function preoperatively, iatrogenic injury or purposeful sacrifice will lead to dysphagia, aspiration, and sometimes increased intracranial pressure from repeated coughing and aspiration [2].

For this reason, some authors emphasize intentional subtotal resections and deliberately leaving residual tumor along the lower cranial nerves (LCNs).

Carlson et al. proposed that the extent of resection, whether total or subtotal, should be tailored to the patient’s perioperative condition and overall life expectancy. In cases of transdural tumor extension with associated brainstem compression, the primary objective should be adequate tumor debulking and neural decompression [6].

### 4.5. Residual-Tumor Management

In advanced tumors (Di2 and Di3) showing extensive involvement of the brainstem, cavernous sinus, cranial nerves III/IV/VI, or the major neurovascular structures, small tumor remnants are generally deliberately left in situ and subtotal or partial removal performed.

There are two primary strategies for managing residual tumor: observation with regular radiological follow-up, since usually these devascularized tumor remnants appear to remain stable over time, and adjuvant stereotactic or fractionated radiotherapy. Radiotherapy may be planned or deferred until radiological evidence of tumor progression is detected.

In our multicenter study, both the patients who underwent adjuvant radiotherapy immediately after partial resection (nine patients) and those whose residue was observed over time (seven patients, three of which showed tumor progression and received targeted radiotherapy) achieved disease control at the end of the follow-up period and did not require salvage surgery. These findings suggest that, in appropriately selected patients, partial resection followed by observation or adjuvant radiotherapy may represent a viable and effective treatment strategy for TJ-PG Class Di paragangliomas. In support of this strategy, Sanna et al. reported that 57.1% of patients with residual tumor showed no growth over time, while 14.3% demonstrated tumor regression [19].

These results support the hypothesis that a residual tumor devascularized during primary surgery may remain stable or regress during the follow-up period, and when regrowth is observed, SRS offers a minimally invasive and effective treatment option, depending on the size and location of the residue. However, despite our experience and the recommendations reported in the literature, larger prospective studies are needed to confirm the long-term outcomes and the optimal management strategy for both stable residual tumors after surgery and for cases of documented progression.

Recently, we employed immunohistochemistry in the form of the Ki-67 proliferation rate, which has been shown to be linked to tumor growth rate. Ki-67 is expressed as a percentage and, while high proliferation rates have been suggested to correlate with malignancy, low (1–2%) and moderate (3–4%) values can be used to guide the choice of management method for the tumor residue. Low proliferation rates can enable close observation of the residue, whereas moderate percentages warrant postoperative radiotherapy. While promising, this needs to be further investigated before basing management decisions on this datapoint [20].

## 5. Conclusions

The management of Di-class TJ-PGs remains challenging due to the absence of standardized treatment guidelines. This multicenter study contributes one of the largest clinical experiences to date, aiming to support and expand the current literature in this field. Treatment options include surgery, stereotactic radiosurgery (SRS), or observation (wait and scan), and the choice should be individualized based on factors such as patient age, comorbidities, lower cranial nerve function, tumor extension, and neurovascular involvement. SRS is best suited for slow-growing tumors without brainstem compression in patients unfit for surgery, while observation may be appropriate for stable, asymptomatic lesions. Surgical resection remains the primary option for symptomatic or progressive tumors, with total resection favored in young, healthy patients, while subtotal resection is preferred in elderly or high-risk individuals, always considering the tumor’s relationship to critical structures, therefore minimizing neurological morbidity. In cases of residual tumor, both radiological surveillance and adjuvant radiotherapy represent effective strategies for long-term control.

## Figures and Tables

**Table 1:** Summary table reporting the Fisch tumor classification, the surgical approaches employed, the extent of resection achieved, and the corresponding cranial nerve outcomes. Facial function is reported according to the House–Brackmann classification.

PATI ENTS	Fisch Class	SURGICAL APPROACH	Stage	REMOVAL	Preop LCN—XII CN Palsy	Postop LCN—XII CN Palsy	Preop VII CN (H.B. Class)	Postop VII CN (H.B. Class)
1	C2 Di1	ITFa	single	partial	X	X	I	I
2	C3 Di2	ITFa	single	partial	none	IX, X, XI	I	I
3	C2 Di2	ITFa	double	total	none	none	I	I
4	C3 Di1	ITFa + far lateral approach	single	total	IX, X, XII	IX, X, XII	I	VI
5	C2 Di1	ITFa	single	partial	none	none	I	I
6	C3 Di1	ITFa	single	partial	none	none	V	VI
7	C3 Di2	ITFa + TL	single	total	IX, X, XI	IX, X, XI	IV	VI
8	C3 Di1	ITFa + TC	single	partial	IX, X, XI	IX, X, XI	IV	VI
9	C3 Di1	ITFa	single	partial	none	IX, X, XI	II	III
10	C3 Di1	ITFa + TL	single	total	IX, X, XI	IX, X, XI	V	VI
11	C4 Di2	ITF + TL	single	partial	IX, X, XI, XII	IX, X, XI, XII	III	VI
12	C3 Di1	ITFa + TL	double	partial	IX, X, XI	IX, X, XI	I	III
13	C3 Di1	ITFa	single	total	IX, X, XI	IX, X, XI	I	III
14	C2 Di1	ITFa + TL	single	total	IX, X, XI	IX, X, XI	II	V
15	C3 Di1	ITFa + TL	single	total	IX, X, XI	IX, X, XI	I	V
16	C3 Di1	ITFa	single	total	IX, X, XI	IX, X, XI	I	III
17	C2 Di1	ITFa	single	total	IX, X, XI	IX, X, XI	II	III
18	C3 Di2	ITFa + TC	single	partial	IX, X, XI, XII	IX, X, XI, XII	I	V
19	C2 Di1	ITFa	double	total	IX, X, XI	IX, X, XI	II	III
20	C3 Di1	ITFa	double	partial	XII	XII	I	II
21	C3 Di1	ITFa + TL	single	total	IX, X, XI, XII	IX, X, XI, XII	VI	VI
22	C3 Di1	ITFa	single	partial	none	none	I	II
23	C1 Di1	ITFa	single	total	IX, X, XI	IX, X, XI	I	II
24	C2 Di1	ITFa	single	total	IX, X, XI	IX, X, XI	I	II
25	C2 Di1	ITFa + TL	single	total	IX, X, XI	IX, X, XI	IV	IV
26	C3 Di2	ITFa + TL	single	total	none	IX, X, XI, XII	I	II
27	C3 Di2	ITFa + TL	double	partial	none	none	I	II
28	C3 Di2	IFTa + TC	double	partial	IX, X, XI	IX, X, XI	VI	VI
29	C3 Di1	IFTa + TL	single	total	IX, X, XI	IX, X, XI	VI	VI
30	C4 Di2	IFTa + TL	single	partial	IX, X, XI	IX, X, XI	VI	VI
31	C2 Di2	ITFa + TL	single	partial	none	none	I	VI
32	C1 Di1	ITFa	single	partial	none	none	I	I

IFTa: Type A infratemporal fossa approach; TL: translabyrinthine approach; TC: transcochlear approach; H.B. class: House–Brackmann classification.

## Data Availability

The original contributions presented in this study are included in the article. Further inquiries can be directed to the corresponding author.
